# COVID-19 Vaccines on TikTok: A Big-Data Analysis of Entangled Discourses

**DOI:** 10.3390/ijerph192013287

**Published:** 2022-10-14

**Authors:** Shaojing Sun, Zhiyuan Liu, Yujia Zhai, Fan Wang

**Affiliations:** 1 Institute for Global Communications & Integrated Media, School of Journalism, Fudan University, Shanghai 200433, China; 2 Management School, Tianjin Normal University, Tianjin 300387, China; 3 School of Information Management, Wuhan University, Wuhan 430072, China; 4 Fudan Development Institute (FDDI), Fudan University, Shanghai 200433, China

**Keywords:** social media, vaccine, TikTok, health communication, big data, China

## Abstract

Focusing on social media affordances and China’s social/political context, the present study analyzed the digital communication practices about COVID-19 vaccines on a popular social media platform—TikTok—which is called DouYin in China. Overall, this study identified five major forces partaking in constructing the discourses, with government agencies and state media being the dominant contributors. Furthermore, video posters demonstrated different patterns of utilizing social media affordances (e.g., hashtags) in disseminating their messages. The top hashtags adopted by state media were more representative of international relations and Taiwan; those by government agencies were of updates on pandemic outbreaks; those by individual accounts were of mainstream values and health education; those by commercial media were of celebrities and health education; those by enterprise accounts were of TikTok built-in marketing hashtags. The posted videos elicited both cognitive and affective feedback from online viewers. Implications of the findings were discussed in the context of health communication and global recovery against the backdrop of the COVID-19 pandemic and Chinese culture.

## 1. Introduction

### 1.1. Background

The ongoing COVID-19 pandemic has wreaked havoc on the world. According to the World Health Organization (WHO), as of 21 January 2022, there were 340,543,962 confirmed COVID-19 cases, including 5,570,163 deaths (see the WHO Coronavirus (COVID-19) Dashboard). In China, by 19 January 2022, the government had reported 4636 deaths and 105,411 confirmed cases (see the homepage of China Center of Disease Control (CDC)). Meanwhile, by 19 January 2022, a total of 9,571,502,663 vaccine doses had been administered worldwide. In China alone, more than 2,947,136,000 doses of COVID-19 vaccines had been implemented by 18 January 2022. As of 1 March 2022, the vaccination coverage rate passed 87% [1]. A recent study using data from 33 countries suggested that COVID-19 vaccination had directly saved at least 470,000 people aged 60 and over, not to mention those lives spared from the indirect effect of vaccination because of reduced transmission [2].

Despite the helpfulness of vaccination on combating COVID-19, increasing vaccination coverage around the world has encountered various challenges. From the beginning, the COVID-19 vaccine has been entangled with politics, science, international relations, economy, etc. Particularly during the early stage of the pandemic, a range of issues—such as vaccine development, vaccine donation, and vaccine diplomacy—were fraught with contestation, controversy, and conflict [3]. The topic of COVID-19 vaccination has not only grabbed legacy media headlines, but also has been frequently presented and discussed on social media platforms.

Considering the vibrancy of discussions about COVID-19 vaccines on social media, such as TikTok, also called DouYin in China, we are interested in a host of questions about the discursive arena of the topic. For example, who were contributing to the discourses about COVID-19 vaccines, particularly during the time when vaccines were in high demand? How did different stakeholders make use of social media affordance to increase the visibility of their content? How were the messages by those content creators received by viewers? Addressing those questions can provide a more comprehensive view of the complex linkages between vaccines, stakeholders, and political/social factors, as social media can reach a much larger size of users compared with legacy media.

The present study seeks to make its contribution in three ways. First, extant research about COVID-19 has mainly focused on Western social media (e.g., Twitter, Facebook) and Western users’ responses to social media messages. In contrast, our study provides a perspective on the pandemic, vaccines, and social media communication in a different culture (i.e., Chinese culture). Second, past research has mostly centered around text-based social media and often excluded rich information based on other modalities (e.g., audio, video) [4]. In comparison, the present study takes into account different modalities with a particular focus on videos and platform-specific affordances. Third, existing studies primarily focused on content analysis of creators’ videos and seldom examined how those videos were received by their viewers [5]. The present study may shed light on the impact of those videos by looking into viewers’ comments on TikTok.

Below, we first provide necessary background knowledge about TikTok, along with relevant literature about social media affordances and Chinese culture; second, we describe our dataset and analytical procedures; third, we present results from a multitude of analytical methods; lastly, we discuss the implications of our findings in relation to social media communication and global recovery from the pandemic.

### 1.2. Literature Review

#### 1.2.1. TikTok and China

The latest report from CNNIC (Chinese network center) suggests that over 1 billion people in China use the internet by the end of June 2022, accounting for roughly 74.4 percent of China’s total population [6]. Researchers and professionals tend to agree that social media can play a big role in crises, such as the ongoing pandemic, because they are more likely to engage the public and provide public-health surveillance [7]. More recently, Schillinger et al. [8] summarized that social media—including TikTok—have become an omnipresent strong force in improving public health by shaping public perceptions, beliefs, norms, and behaviors.

TikTok, a platform highly popular among young users, announced that its monthly active users had exceeded 1 billion by the end of September 2021 [9]. In China, the number of monthly active TikTok users has already passed 0.6 billion, with daily video searching inquiries exceeding 0.4 billion [10]. TikTok has been actively involved in China’s national combat against the pandemic. An official report [9] indicated that COVID-19-related videos racked up more than 42.3 billion views on TikTok and more than 16 million people turned to TikTok to keep themselves updated on the pandemic. Moreover, TikTok videos featuring doctors/nurses received more than 1 billion likes and positive comments from viewers.

To better understand the operation of TikTok, it is necessary to examine its linkage to China’s political and societal system. On this note, one of the most revealing buzzwords is “positive energy” (“正能量”in Chinese), which has been prevalent in Chinese political discourses and media reporting since 2012. The term has been frequently deployed by Chinese governments to advocate for disseminating information supporting the ruling authorities and moral behaviors. Chen et al. [11] observed that, as of June 2018, over 500 Chinese government agencies opened TikTok accounts to post videos featuring positive energy, gathering over 1.6 billion views. Furthermore, video length, title length, use of interactive questions, and content type (e.g., expressing gratitude for frontline workers’ services) significantly influenced the level of citizen engagement with those videos. Similarly, Zhu et al. [12] found that nearly half of the Provincial Health Committees (PHCs) in China have opened TikTok accounts to engage with local residents and promote health-related knowledge. Zhu et al. found that those videos featuring cartoon and documentary-style content were viewed most frequently.

#### 1.2.2. Social Media Affordances

The power of social media is rooted in the diverse and rich affordances they provide. Social media affordances refer to the relational possibilities/capabilities for communicative actions that emerge from the interactions between users and features of media [13]. Treem and Leonardi [14] illustrated four major affordances—including visibility, editability, persistence, and association—that are pivotal in driving and structuring digital communication practices and processes. Those general and abstract affordances—which reflect the complex and dynamic relationships between digital media, users, and the context—typically correspond to concrete functions and designs of the platform. For example, TikTok’s algorithm recommendation and meta-data (e.g., number of likes) make some posts more visible than others; the option to alter or delete a video after posting speaks to editability; the sustainability of profile information reflects persistence; the use of hashtags captures the association between users, content, and topics.

It is argued that affordance is an enlightening concept because it falls into neither technological determinism nor subjective constructivism. In other words, the concept accentuates a relational perspective and acknowledges user’s agency in interacting with media and the environment. As Bailey et al. [15] contended, users perceive and experience affordances in light of their goals and the possibilities to accomplish their goals, and such affordances are often enabled or constrained by the medium’s “rules, grammar, and structure” (p. 252). That said, in reality, users may actively take advantage of certain affordances enabled by the medium to achieve their goals. Certainly, users’ activity in actualizing specific affordances is contingent on their interactions with the medium and the social/cultural context.

A body of studies has explicated how different kinds of social media affordances link to communication processes and effects. For instance, Sun et al. [13] reported that the social media platform Weibo had assisted early COVID-19 patients under lockdown to seek help and access medical resources. Specifically, those users employed Weibo-afforded hashtags to form a support-provision online community and used the @ function to reach out to media and medical resources. Meanwhile, online readers actively participated in endorsing, forwarding, and commenting on support-seekers’ posts, to keep the communication process unfolding over a long period of time. Studying online political movements, Pond and Lewis [16] found that both hashtag usage and account status were vital in shaping message crafting and sense making on Twitter. Celebrity accounts were more influential in framing Twitter discourses than other accounts. Further, the authors found that a more emotive but less noted hashtag had exercised a larger and much more complex effect on the online movement than other popular hashtags. This emotive effect comports with the affective characteristics of social media users dubbed as affective publics by Papacharissi [17].

#### 1.2.3. Linking Users, Affordances, and COVID-19 Vaccines

Past research has examined the relationship between different vaccines (e.g., HPV, Zika, influenza) and communication processes involving various social media platforms, such as Twitter, Instagram, and TikTok. Limaye et al. [18] reviewed past studies on social media and vaccine acceptance, and the authors found that a range of social-media-related factors—such as who delivers the message, how a message is framed, and online network structure—affect individuals’ vaccine decision-making process. Recently, Luo et al. [19] studied public discussions about the COVID-19 vaccines on two social media platforms—Twitter and Chinese Weibo—and found significant discrepancies between the two platforms. On Twitter, users tended to disclose personal experiences of vaccination and voice anti-vaccine attitudes; on Weibo, users tended to express positive sentiments toward the vaccines and respect for authorities. Relatedly, Zhang et al. [4] analyzed 156,223 COVID-19-vaccine-related Weibo posts and observed that public approval toward the vaccines fluctuated over time. Furthermore, a regional outbreak or reported incident affected public willingness to get vaccinated. Monselise et al. [20] analyzed a large corpus of tweets spanning a 60-day time period and found that access to vaccines was a major public concern expressed in the tweets. Furthermore, fear and joy were the top two most prevalent emotions characterizing the tweets.

Song et al. [21] examined four types of affordances (livestreaming, searching, meta-voicing, and recommending) by TikTok and found that users’ experience (immersion, social presence, and credibility perception) was positively associated with the different affordances. In more detail, Civila and Jaramillo-Dent [22] described a variety of affordances unique to TikTok—such as the algorithmic *for you* feed which increases a video’s visibility, diverse audio tracks that render a video more performative, and unique emoticons that help enrich discursive expression. Digging into the recommendation system, Schellewald [23] summarized six communicative forms of TikTok: comedic, communal, documentary, explanatory, interactive, and meta forms. All these forms suggest that certain features or functions of TikTok afford users to create contents that are likely to go viral on the platform. Schellewald argued that TikTok is more than an entertainment platform, and that the medium creates an ambience that immerses users to create a variety of cultural artefacts. To understand the rich discourses about COVID-19 vaccination on TikTok, we propose the following questions:

RQ1: What is the general landscape of COVID-19-vaccine-related videos, video producers, and focal topics on TikTok?

RQ2: Are there differences in the strategies of promoting video messages (e.g., using hashtags) by different types of video producers?

In China, local governments are responsible for monitoring, managing, and coping with public-health crises. Gao and Yu [24] explained the role of local governments as “meta governance” to coordinate multiple stakeholders. Limaye et al. [18] pointed out that extant research on social media and vaccination excessively focused on high-income countries and Western social media platforms. As a result, there is a lack of knowledge about harnessing social media to advance vaccination coverage in middle- and low-income countries. China, housing an enormous population and an expansive community of social media users, serves as a rich and unique research context for exploring social media communication and COVID-19 vaccination. More importantly, social media, such as TikTok, have ushered in new modes of communication, characterizing connection, participation, interaction, and co-creation between users. To look into the aforementioned new communication modes, we raise the third research question as below:

RQ3: What are the characteristics of viewers’ cognitive and affective responses to those vaccine-related videos? Are there differences in commenters’ responses to the videos posted by different types of TikTok accounts?

## 2. Methods

**Figure 1:**
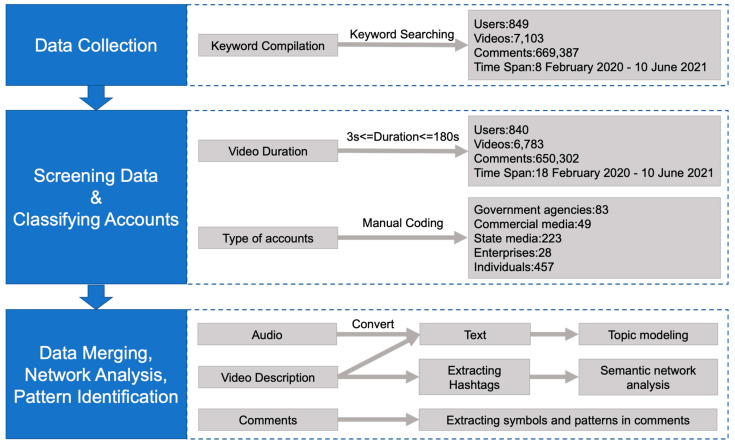
The procedure of data processing.

The overall procedure of data processing is shown in Figure 1. Due to the API constraints, the research team accumulated TikTok data spanning from 18 February 2020 to 10 June 2021, which, however, covers the most important stages and events about COVID-19 vaccines in China. Ten hashtags (e.g., #COVID-19 vaccine, #COVID-19 vaccination) compiled by the research team in a pilot study were used to identify suitable videos. Overall, 7103 videos were collected over the data collection period. We filtered out videos that were shorter than 3 s and those that were longer than 180 s. Finally, 6783 videos were retained along with 650,302 comments to those videos. The research team first randomly sampled 800 videos from the built database and then created an initial typology of video posters, including government agencies, state media, commercial media, enterprises, and individual accounts [25]. Our pilot coding showed that very few scientists and medical experts set up their own TikTok accounts, though those experts were often featured in the videos posted by other accounts.

First, we analyzed the temporal distribution of videos, the number of videos posted by users, and the video topics by applying the top2vec modeling method. Second, we conducted a social network analysis to construct hashtag co-occurrence networks for different types of video producers. Third, we employed natural language processing (NLP) to summarize the most prevalent phrases in viewers’ verbal comments, as well as representative emoticons that viewers used to express their emotions.

**Topic extraction.** To obtain the full range of topics contained in the data, we applied the Top2Vec method [26], which uses joint document and word semantic embeddings and hierarchical-density-based spatial clustering to identify topic vectors in the corpus. For preprocessing, we first extracted the audio contained in all TikTok videos and then converted them into texts using the voice dictation service provided by the IFLYTEK open platform, which has an average of 99.4% accuracy for converting Chinese speech to text [27]. The title of each video was then combined with the text-converted speech to generate a video-description text. Next, we used the Jieba toolkit, which is specifically designed for Chinese natural language processing, to perform word segmentation and part-of-speech tagging on this corpus. To reduce the noise of functional words, we only kept those nouns, adjectives, and verbs containing at least two Chinese characters.

Top2Vec is a topic modeling approach that uses joint documents and word semantic embeddings to discover topic vectors [26]. It consists of algorithms that automatically find dense clusters in a collection of input documents with the assumption that semantically similar documents constitute topics. The modeling process of Top2Vec is as follows.

1. Create a joint semantic embedding using doc2vec [28]. The distance between the document vector and the word vector represents the semantic association. In the embedding space, semantically similar documents are placed together and words should be close to the documents they describe. This spatial representation of words and documents is called semantic space.
2. Reduce the dimensionality of the document vector using UMAP [29]. Since the semantic embedding space created by doc2vec is a high-dimensional space in which the document vectors are very sparse, dimensional reduction helps to discover dense regions. UMAP is a stream learning technique for dimensional reduction with a strong theoretical foundation, which preserves local and global structure and is able to scale to very large datasets.
3. Find dense document clusters and group semantically similar documents using HDBSCAN [30]. HDBSCAN is used to find dense regions of document vectors as it is designed to handle noisy and variable density clusters. HDBSCAN assigns a label to each dense cluster of a document vector and a noise label to all document vectors that are not in a dense cluster.
4. Calculate the centroid in the original dimensional space. The dense clusters of documents identified by HDBSCAN correspond to positions in the original semantic embedding space. The use of UMAP and HDBSCAN can be considered as a process that labels each document in the semantic embedding space with a noise label or the label of the dense cluster to which it belongs. The topic vector is calculated as the mean of all document vectors in the same dense cluster.
5. Identify topic words. The words closest to the topic vector can be considered as the words most similar to all documents in the dense region, since the topic vector is the center of mass of the region. These words are then used to summarize the common themes in the documents in the dense region.

The advantage of Top2Vec is reflected by its capability of identifying the number of clusters. Density-based clustering requires no intervention by manually setting the number of clusters. Furthermore, document and word embeddings provide more generality than LDA topic modeling [31], such as comparing documents across subsets via model alignment. Furthermore, this method does not require removing stop words or prior knowledge of existing topics to produce a good topic model. Once the model is generated, it will provide various information such as topic size, topic words, topic number and topic score, etc. Topic size denotes the number of documents that are most similar to each topic and for each topic, the top 50 words are returned in the order of descending semantic similarity to the topic. The topic score represents the cosine similarity of each topic to the related keyword. The higher the topic score, the more representative the keyword is of the topic. The model returns the cosine similarity between each video and each topic. For example, the similarity between video A and topics 1, 2, and 3 is 0.5, 0.3, and 0.2, respectively, meaning that video A belongs to topic 1. By calculating the similarity between all videos and topics, we obtained the cross-time distribution of each topic based on the posting time of each video.

We chose the “deep learning” parameter when creating topic models with our own data. The Top2Vec model was trained on the TikTok dataset and generated 110 initial topics. We further performed hierarchical topic reduction by iteratively merging similar topics until reaching the desired number of topics. The original structure of the initial topics was preserved and can be queried to determine which original topics contain after-reduction topics for further inspection. As a result, our analysis reduced the number of topics to 10 compact meta-topics. Two coders assessed representative keywords and named topics independently, with an initial coding agreement of 82% and consistency reliability of 0.9. The two coders then compared and discussed the codes one by one until they reached a complete consensus.

**Topic distribution.** The results of Top2Vec identified the associated topics for each video. Considering the release dates of the videos, we counted the number of videos each topic contains and used river graphing to represent the topic’s monthly distribution over time. The width of each branch represents the proportion of the topic in the corresponding month. For example, if the number of videos corresponding to topics 1, 2, and 3 in March 2020 is 20, 30, and 50, respectively, then the corresponding distribution for topic 1 in that particular month is 20%.

**Hashtag co-occurrence network.** First, we extracted all the hashtags contained in the video titles. Next, we removed the common hashtags (e.g., #COVID-19) contained in all videos and constructed the hashtag co-occurrence networks for each type of video poster—including government agencies, state media, commercial media, enterprises, and individual accounts. We used Gephi [32] and the Force Atlas 2 layout algorithm [33] to visualize the networks.

## 3. Results

### 3.1. RQ1: Descriptive Statistics of Videos, Producers, and Topics

The distribution of video posters was summarized as below: government agencies, 84 accounts (9.89%), posting 1164 videos, accounting for 16.39% of the total videos; state media, 224 accounts (26.38%), posting 4614 videos (64.96%); commercial media, 51 accounts (6.01%), 209 videos (2.94%); enterprises, 28 accounts (3.30%), 107 videos (1.51%); and individual users, 462 accounts (54.42%), 1009 videos (14.21%). Overall, state media was a dominant poster, with each media account posting about 21 videos on average. In contrast, individual users accounted for nearly 55% of the total posters but, on average, each account only posted about two videos.

The Top2Vec method generated six major vaccine-related topics. For each topic, the research team carefully reviewed the top 50 representative keywords and reached a consensus on naming the topic. The topics and their respective proportions in the videos were as below: vaccination access and services (34.26%), vaccine donation and international relations (16.41%), positive energy and mainstream values (16.15%), vaccination precautions and advice (11.92%), vaccine research and development (11.75%), updates on COVID-19 outbreaks (9.51%) (see Table 1 for representative keywords). Interestingly, for state media, the topics were distributed roughly in a balanced manner. In contrast, for other types of accounts, there were striking discrepancies in proportions across different topics. For instance, for government agencies, topics such as positive energy and cautions about fraud were more prevalent, whereas topics about vaccination precautions were less frequent.

Furthermore, across the five types of poster accounts, the top videos posted by state media accumulated much higher viewer responses, as compared to those by other accounts. The top videos mainly featured China’s success in developing COVID-19 vaccines and the availability of those vaccines to the general public.

### 3.2. RQ2: Hashtag Use and Semantic Networks

Results showed significant differences in the type and frequency of hashtags used by different accounts (Table 2). The top hashtags used by state media were more related to politics and media, such as #Taiwan, #latest-news, #America, etc.; government agency accounts frequently used hashtags associated with the names of government agencies and the pandemic, such as #State-Council, #Health-China, #pandemic-control; individual users often employed hashtags bearing on science education, such as #medical-science, #health-education; commercial media accounts implemented more hashtags featuring celebrities and health, such as #Zhang-Wenhong and #health; enterprise accounts incorporated more hashtags pertaining to businesses, such as #micro-insurance and #vaccine-stock. Our results also showed that several hashtags appeared frequently across different accounts. For instance, the popular hashtag #Zhong-Nanshan featured a medical expert who gained his fame during China’s combat against several massive epidemics, including SARS and H7N9 crises.

**Figure 2:**
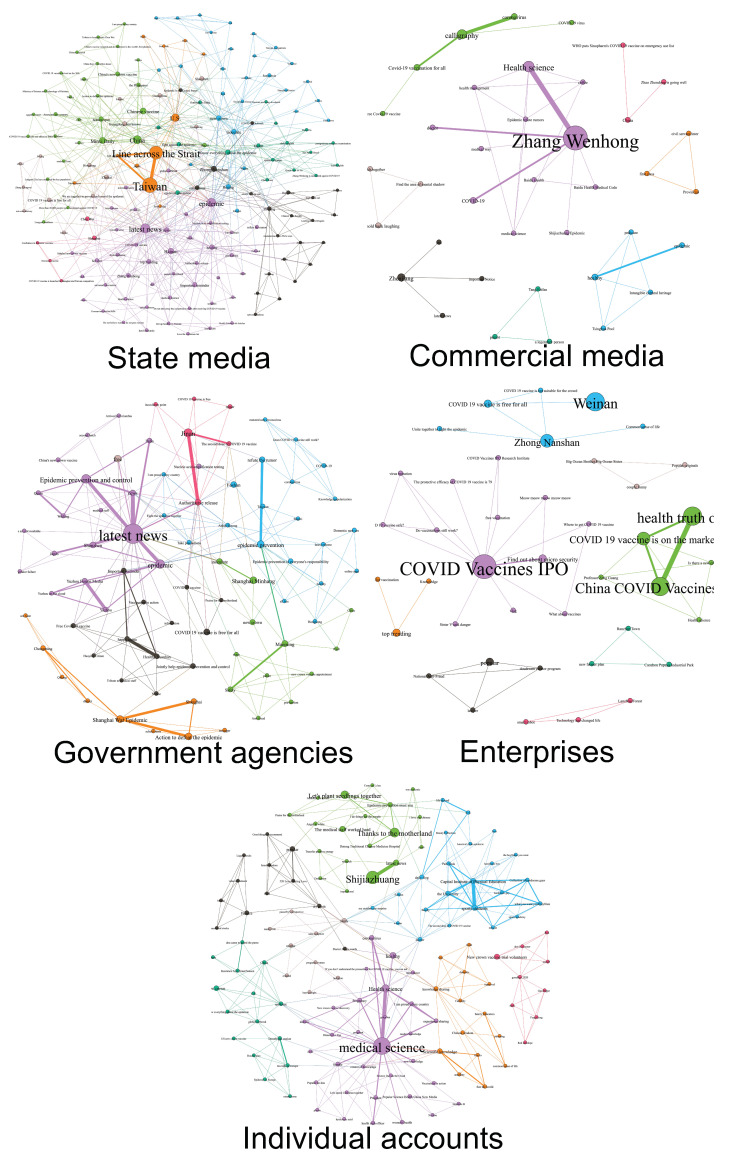
Hashtag co-occurrence network by account types.

Furthermore, media accounts used far more hashtags than other types of accounts. We examined the community partition of hashtag networks by account type (see Figure 2). For state media accounts, the top two identified communities were the following: news updates, accounting for 8.57%, represented by hashtags such as #latest-news, #news, and #hot-news; science about coronavirus, accounting for 7.72%, represented by #coronavirus, #medical-science, and #Zhong-Nanshan. For the accounts of government agencies, the top hashtag communities were: updates on the pandemic, accounting for 10.36%, represented by #pandemic-control, #pandemic, and #news; call for vaccination, accounting for 5.82%, represented by #free-vaccine and #first-shot. For commercial media, the top two communities were: science education, accounting for 12.12%, represented by #Zhang-Wenhong and #health-education; vaccination, accounting for 5.05%, represented by #free-vaccine and #collective-vaccination. For individual accounts, the top two communities were: medical education, accounting for 11.47%, represented by #medicine-edu and #medical-knowledge; positive energy, accounting for 6.04%, represented by #praise-homeland and #we-will-win. For the accounts of enterprises, the top two communities included: exemplar enterprises, accounting for 17.33%, represented by the hashtag #wei-bao; call for vaccination, accounting for 8%, represented by #free-vaccine and #caution-vaccine.

### 3.3. RQ3: Viewers’ Response to Featured Videos

Our analysis demonstrated significant differences in viewers’ comments across the type of video posers’ accounts (Table 3). On average, videos by individual accounts were the most frequently commented, with a median of 35 comments and a maximum of 7152. In contrast, videos by government agencies were least frequently commented, with a median of 9 and a maximum of 308 comments. Our results also showed that viewers actively used emoticons and emojis to express their feelings and emotions. Among all the emoticons, thumbs-up was the most frequently presented, followed by symbols signifying tears and clapping. In terms of verbal language, medical words (e.g., vaccine, inoculation, second dose) and positive energy (e.g., motherland, hope, thanks) appeared most frequently. Interestingly, across all accounts, we identified seven kinds of response patterns: verbal text only, text + emoticon, text + @, @ only, emoticon only, emoticon + @, a mixture of all the aforementioned elements. Of these patterns, nearly half of the analyzed comments adopted a pattern of verbal text only. More than 30% of the total comments adopted a pattern of text combined with emoticon. In comparison, viewers’ comments to the videos posted by enterprises were more likely to involve the use of @, a directed communication pattern, though the percentage was only slightly above 10%.

We also took a look at those comments that received the highest endorsement from other viewers. Results showed that the most popular comments can be roughly classified into the following categories: expressing national pride (e.g., “Bravo, this is China.” “Thank homeland, thank researchers!”), humor or self-mocking (e.g., emoticons), personal value (e.g., “Get your umbrella even when it is a sunny day.”), questions, or suggestions about vaccination (e.g., “Can I have COVID-19 vaccine even if I had rabies vaccine recently?”). In general, those comments were brief and the majority of them had a length below ten Chinese characters.

## 4. Discussion

### 4.1. Major Findings

To our knowledge, this is the first study to employ a big-data approach to examine digital communication about the COVID-19 vaccine on TikTok. Recent studies have mostly focused on how text-based social media platforms communicate COVID-19 vaccination. For instance, Luo et al. [19] identified a few discussion themes cutting across Twitter and Chinese Weibo, including domestic policies about vaccination, global pandemic trends, COVID-19 variants, and susceptible groups. In contrast, our study identified more diverse themes about COVID-19 vaccines on TikTok. Most importantly, our results showed that vaccination is not a mere medical or scientific issue; rather, it is intricately entangled with politics, culture, and social norms. As Luo et al. argued, culture characteristics (e.g., power distance, uncertainty avoidance) are a main driver for the cross-platform differences in online discussions about the vaccine.

A key finding of our study is the five different types of stakeholders who have been active in posting videos about COVID-19 vaccines, including state media, government agencies, commercial media, enterprises, and individual accounts. Among all the accounts, state media was the most active and influential video poster, as they posted the vast majority of the relevant videos and received an overall larger amount of feedback (e.g., Likes, forwarding) from viewers. Interestingly, very few scientists and medical experts opened personal accounts on TikTok; instead, they were frequently quoted or interviewed in videos posted by government accounts or media accounts.

The second major finding is that hashtag use served as a vital communication strategy for stakeholders to publicize their videos. State media were the stakeholder that used much more diverse hashtags than other accounts. Furthermore, different stakeholders demonstrated preferences for varying types of hashtags, though they did share some common popular hashtags. State media and government agencies used hashtags bearing on politics and mainstream ideology most often; commercial media adopted hashtags about celebrities most frequently; individual accounts frequently employed hashtags about science and education.

The third significant finding is about the active participation of social media users in viewing vaccine-related videos. Not only did users cognitively participate but also affectively participate in responding to those featured videos. Viewers expressed their feelings, emotions, and sentiments by creatively utilizing various emoticons and emojis; also, viewers provided meaningful questions or advice. For example, some viewers suggested in their comments that the government set up more stations or booths nearby their living communities so that residents can easily get vaccinated. More interestingly, such suggestions prompted further interaction and exchange of opinions between viewers of the same video.

### 4.2. Theoretical Implications

Our research findings hold rich theoretical implications for social media research. First, our study testifies to the complex interactive relationship between traditional mainstream media (e.g., newspapers, TV stations) and emerging social media platforms. Traditional media have become a powerful group of content producers on TikTok, illustrative of the strong trend of media convergence in the contemporary media society. In other words, the boundary between traditional media and social media is becoming blurry; different media (both traditional and newer ones) are embedded within each other. Researchers have created various terms—such as “polymedia,” “convergent media,” or “omni-media”—to describe the ever-evolving media landscape. Madianou and Miller [34] noted that social media have provided a communicative environment of affordances rather than a catalog of discrete technologies. Advocating for a theory of polymedia, the authors argued that such popular social media carry and reflect a new relationship between the social and the technological. Our study has added further insight into the polymedia theory by demonstrating how complex media, multiple stakeholders, and rich discourses interact with each other to construct a vibrant communication environment.

Second, our findings on the use of hashtags shed light on the powerful theoretical construct affordances. Rathnayake and Suthers [35] contended that hashtags can be viewed as a kind of media affordance because of the following: (a) social media afford its users to create hashtags and (b) user actions (e.g., discussion, engagement measurement) emerge from the use of hashtags. Our findings suggest that hashtag use is connected to those video posters’ political positions, brandings needs, and communication goals. Rauschnabel et al. [36] uncovered ten different motivations driving social media users to use hashtags, including amusing, designing, organizing, trend engaging, bonding, conforming, inspiring, summarizing, reaching, and endorsing. The authors further showed that these motivations vary across social media platforms and link to sophisticated online behavior. The differing patterns of using hashtags indicate that concrete technological affordances (e.g., hashtags) may carry different meanings depending on users’ motivations and social cultures. The multi-purposeful usage of hashtags may inform future theorization of social media affordances, especially video-based and algorithm-driven media [14].

### 4.3. Implications for Global Recovery

Practically, our research findings may inform global recovery from the pandemic on improving communication, building resilience, and nudging action. First, throughout the pandemic, stakeholders have made use of video-based social media in differential manners. As for communication about COVID-19 vaccination, state media and government agencies are more active communicators than scientists and health experts. One plausible explanation is that most Chinese scientists are not familiar with or do not possess the skills of using TikTok. Indeed, according to the most recent statistics, the vast majority of Chinese TikTok users are people aged from 18 to 30 [10]. Governments and non-profit organizations can consider investing personal resources to help scientists set up and manage social media accounts for disseminating science and promoting public health.

Second, social media are a valuable and powerful communication channel to mobilize people and build resilient communities. Our study has shown that video posters, be it state media or individual accounts, have a good grasp of so-called positive energy—which is state endorsed but also features lighthearted or amusing media content, depicted as playful patriotism by Chen et al. [11]. Such playful patriotism has proven to be influential in galvanizing people and engaging people in taking preventive measures, including vaccination against COVID-19. That being said, governors, researchers, and health-promotion professionals should be aware of the unique ambiance, audience, and affordances of popular media such as TikTok. Employing diverse social media affordances (e.g., hashtags, music) may assist in communicating pro-social values, strengthening communal solidarity, and facilitating cooperative efforts for global recovery.

Third, compared to text-based platforms, video-based social media such as TikTok have the power to vividly present what is going on at grassroots levels. The videos analyzed in the present study captured diverse, immediate, broad, and vivid scenes about local communities and residents. It is quite common for viewers to identify videos that are close to them in terms of both physical and psychological distance. Research has shown that socially immediate videos are more likely to influence people, both cognitively and affectively [11,25]. Global recovery efforts should take advantage of the power of multi-modality communication through social media, to help people relate society-level recovery measures to their immediate living contexts. By making communication more relatable, recovery campaigns or interventions are more likely to nudge people into action and cooperation.

Fourth, our study suggests that to better wield the power of social media to combat the pandemic, it is necessary to link social media affordances to contextual and cultural factors. In other words, social media do not operate in a vacuum, but rather interact with politics, values, traditions, and lifestyles. For instance, the Chinese political system may exert a significant impact on the popularity of videos featuring positive energy on TikTok, as Chinese media—including social media in China—are supervised to comply with the rules and regulations enacted by the government.

### 4.4. Limitations and Future Directions

The present study has several limitations that warrant further attention. First, our study only analyzed the data from a single social media platform (i.e., TikTok), though nowadays many people use multiple social media platforms in their daily lives. Future research should consider integrating data from multiple platforms and explore how those platforms interact, enhance, or mitigate each other. Second, merely relying on the publicly available data, the current study cannot detect exactly what factors drive stakeholders to post videos and what are the underlying values guiding their selection and usage of affordances such as hashtags. Future research can survey or interview video producers and dig into their in-depth motivations for posting. Third, due to the restriction of TikTok, our data do not cover all the comments under each video, neither are commenters’ personal profiles accessible for downloading. Future research may try alternative methods (e.g., experiments) to remedy the weakness of the present study.

## 5. Conclusions

Our findings are useful for developing communication strategies through social media, particularly video-based ones, to disseminate authoritative health information and build trust between different stakeholders. Despite the continued impact of the pandemic, anti-vaccine voices and movements are thwarting the progress of global recovery. It should be noted that existing research has mainly focused on the dark side of social media in COVID-19 vaccination, such as vaccine hesitancy, misinformation, and anti-vaccine movements. As a result, little research has investigated how to use social media proactively for vaccination uptake. Research has corroborated that social media-based interventions are effective in affecting people’s attitudes toward vaccines [37]. Put differently, social media do hold tremendous potential to affect and moderate the outcomes of various pandemic response measures, such as vaccination. More concretely, as Leng et al. [38] pointed out, to improve COVID-19 vaccine uptake, health authorities should proactively explicate the side effects of vaccines, promote vaccine effectiveness, and communicate local vaccine coverage in a timely manner.

Our research has demonstrated an urgent need to revisit the relationship between social media and health communication during times of public-health crises, such as the ongoing pandemic. Governors, scientists, media professionals, enterprises, and average citizens are all significant participating forces in the arena of social media communication. Thus, understanding those stakeholders’ views and discourses should be an important step along the long journey toward global recovery.

## Figures and Tables

**Table 1:** Major topics of TikTok videos based on topic modeling.

Topics	Keywords	Topical Ratio
vaccination access & services	doses, accumulated vaccines, first dose, appointment, vaccination, residents, medical care, clinics, service, fees, prevention, population	34.26%
vaccine donation & international relations	delivery, airport, Taiwan, WHO, Sinovac Co., China, president, developing countries, Serbia, Philippines, donation, Hungary, USA, global, Chile, Cambodia, Chile, procurement orders	16.41%
positive energy & mainstream values	good wishes, homeland, deep love, flower, human world, romance, pride, fortunate, home country, sense of security, stars, the Sun, thanks, I love you	16.15%
vaccination precautions & advices	advice, pregnancy, science education, fever, acute illness, pregnant woman, chronic illness, side effect, breast feeding, antibody, allergy, fetus, immunity, anemic	11.92%
vaccine research & development	clinical trial, Chen Wei (a scientist), vaccine via aerosol, big news, approval, FDA, vaccine productivity, on market, research team, Sinovac Co.	11.75%
updates on COVID-19 outbreaks	avoid one-size-fits-all, cotton swab, forceful, nursing home, infected, outbreak, test positive, asymptomatic, new cases, confirmed infection, nucleic acid test, health ministry, pandemic news	9.51%

**Table 2:** Top hashtags used by different types of accounts.

User Category	Rank	1	2	3	4	5	6	7	8	9
State media	Hashtag	#Taiwan	#update-on-the-straits	#latest-news	#China	#America	#Taiwan-straits	#China-vaccine	#Fujen-daily	#Hainan
	Count	198	169	110	85	80	80	55	53	47
	Ratio	4.19%	3.57%	2.33%	1.80%	1.69%	1.69%	1.16%	1.12%	0.99%
Government agencies	Hashtag	#latest-news	#State-Coucil	#Health-China	#pandemic-control	#pandemic	#Jinan	#Zhong-nanshan	#defeat-pandemic	#Chang-ge
	Count	62	27	24	22	20	18	16	15	15
	Ratio	5.04%	2.19%	1.95%	1.79%	1.62%	1.46%	1.30%	1.22%	1.22%
Individual users	Hashtag	#health-education	#ShiJiaZhuang	# Thank motherland	#south-health	#vaccination-together	#pandemic-in-France	#health-science	#health	#thanks-to-doctors
	Count	66	49	28	24	22	22	21	14	13
	Ratio	5.64%	4.18%	2.39%	2.05%	1.88%	1.88%	1.79%	1.20%	1.11%
Commercial media accounts	Hashtag	#Zhang-wenhong	#health-education	#books	#Zhenjiang	#Zhong-nanshan	#vaccine-for-everyone	#coronavirus	#health	#pandemic
	Count	44	16	11	7	5	4	4	4	4
	Ratio	21.26%	7.73%	5.31%	3.38%	2.42%	1.93%	1.93%	1.93%	1.93%
Enterprises accounts	Hashtag	#micro-insurance	#Weinan-Shaanxi	#China-vaccine-stock	#health-truth	#vaccine-stock	#Zhong-nanshan	#vlog	#his-potentials	#trending
	Count	8	6	6	6	4	4	4	4	3
	Ratio	6.90%	5.17%	5.17%	5.17%	3.45%	3.45%	3.45%	3.45%	2.59%

**Table 3:** Descriptive statistics of comments to videos by video-producer type.

Category	State Media	Government Agencies	Enterprises	Individuals	Commercial Media
# comments	166,477	26,970	16,741	411,478	28,636
Min #	1	1	1	1	1
Median	13	9	7	35	20
Max #	4763	308	4731	7152	4910
Video count	3874	859	95	889	174
User count	223	83	25	412	45
Top Emoticon	👍 40.54%, 🤦 12.29%, 👏 9.07%, 🌹 4.91%, 🙏 3.49%, 🤞 3.33%, 😁2.86%	👍 43.42%, 👏 10.04%, 🤦 9.27%, 🌹 5.45%, 🤞 4.74%, 🙏 3.02%, 😁 2.82%	🤦 30.31%, 👍 17.48%, 🐶 7.72%, 😁 4.64%, 🌹 2.90%, 👏2.65%, 🤞 2.51%	👍 29.89%, 🤦 20.05%, 🌹 6.21%, 👏 4.87%, 🤞 4.43%, 😁 3.20%	👍 25.79%, 🤦 21.40%, 😁 6.12%, 👏 5.80%, 🤞 4.44%, 🌹 4.39%
Text only	51.46%	54.20%	39.65%	47.08%	40.19%
Text & emoticon	35.68%	32.18%	31.90%	32.86%	38.72%
Text @	2.07%	1.79%	7.66%	5.49%	5.28%
@	3.58%	3.52%	11.29%	6.41%	7.25%
Emoticon only	5.34%	6.11%	2.07%	3.43%	3.25%
mixture	1.37%	0.77%	5.72%	3.82%	4.13%
Emoticon @	0.50%	0.42%	1.67%	0.89%	1.18%

## Data Availability

Data are obtained from Douyin website https://www.douyin.com/ (accessed on 9 October 2022).

## References

[1] (2022). Chinese Center for Disease Control and Prevention. https://www.chinacdc.cn/.

[2] Meslé M.M., Brown J., Mook P., Hagan J., Pastore R., Bundle N., Pebody R.G. (2021). Estimated number of deaths directly averted in people 60 years and older as a result of COVID-19 vaccination in the WHO European Region, December 2020 to November 2021. Eurosurveillance.

[3] Su Z., McDonnell D., Li X., Bennett B., Šegalo S., Abbas J., Xiang Y.T. (2021). COVID-19 vaccine donations—Vaccine empathy or vaccine diplomacy? A narrative literature review. Vaccines.

[4] Zhang S., Pian W., Ma F., Ni Z., Liu Y. (2021). Characterizing the COVID-19 infodemic on Chinese social media: Exploratory study. JMIR Public Health Surveill..

[5] Eriksson M., Åkerlund M. (2022). Through a white lens: Black victimhood, visibility, and whiteness in the Black Lives Matter movement on TikTok. Inf. Commun. Soc..

[6] (2022). The 50th Statistical Report on the Development of the Internet in China. http://www.cnnic.net.cn/NMediaFile/2022/0926/MAIN1664183425619U2MS433V3V.pdf.

[7] Kass-Hout T.A., Alhinnawi H. (2013). Social media in public health. Br. Med. Bull..

[8] Schillinger D., Chittamuru D., Ramírez A.S. (2020). From “infodemics” to health promotion: A novel framework for the role of social media in public health. Am. J. Public Health.

[9] Kemp S. Digital 2021 October Global Statshot Report. https://datareportal.com/reports/digital-2021-october-global-statshot.

[10] (2021). DouYin 2021 Data Report. https://cloud.tencent.com/developer/article/1949920.

[11] Chen X., Valdovinos Kaye D.B., Zeng J. (2021). # Positive Energy Douyin: Constructing “playful patriotism” in a Chinese short-video application. Chin. J. Commun..

[12] Zhu C., Xu X., Zhang W., Chen J., Evans R. (2020). How health communication via TikTok makes a difference: A content analysis of TikTok accounts run by Chinese Provincial Health Committees. Int. J. Environ. Res. Public Health.

[13] Sun S., Seo M., Wang F., Liu Z. (2021). Social Support and Connective Affordances: Examining Responses to Early COVID-19 Patient Support Seeking on Microblogs. J. Broadcast. Electron. Media.

[14] Treem J.W., Leonardi P.M. (2012). Social media use in organizations: Exploring the affordances of visibility, editability, persistence, and association. Commun. Yearb..

[15] Bailey R.L., Read G.L., Yan Y.H., Liu J., Makin D.A., Willits D. (2021). Camera point-of-view exacerbates racial bias in viewers of police use of force videos. J. Commun..

[16] Pond P., Lewis J. (2019). Riots and Twitter: Connective politics, social media and framing discourses in the digital public sphere. Inf. Commun. Soc..

[17] Papacharissi Z. (2016). Affective publics and structures of storytelling: Sentiment, events and mediality. Inf. Commun. Soc..

[18] Limaye R.J., Holroyd T.A., Blunt M., Jamison A.F., Sauer M., Weeks R., Gellin B. (2021). Social media strategies to affect vaccine acceptance: A systematic literature review. Expert Rev. Vaccines.

[19] Luo C., Chen A., Cui B., Liao W. (2021). Exploring public perceptions of the COVID-19 vaccine online from a cultural perspective: Semantic network analysis of two social media platforms in the United States and China. Telemat. Inform..

[20] Monselise M., Chang C.H., Ferreira G., Yang R., Yang C.C. (2021). Topics and sentiments of public concerns regarding COVID-19 vaccines: Social media trend analysis. J. Med. Internet Res..

[21] Song S., Zhao Y.C., Yao X., Ba Z., Zhu Q. (2021). Short Video Apps as a Health Information Source: An Investigation of Affordances, User Experience and Users’ Intention to Continue the Use of TikTok. Internet Res..

[22] Civila S., Jaramillo-Dent D. (2022). # Mixedcouples on TikTok: Performative Hybridization and Identity in the Face of Discrimination. Soc. Media+ Soc..

[23] Schellewald A. (2021). Communicative forms on TikTok: Perspectives from digital ethnography. Int. J. Commun..

[24] Gao X., Yu J. (2020). Public governance mechanism in the prevention and control of the COVID-19: Information, decision-making and execution. J. Chin. Gov..

[25] Chen Q., Min C., Zhang W., Ma X., Evans R. (2021). Factors driving citizen engagement with government TikTok accounts during the COVID-19 pandemic: Model development and analysis. J. Med. Internet Res..

[26] Angelov D. (2020). Top2vec: Distributed representations of topics. arXiv.

[27] Cao C., Wang G. (2020). Evaluation of intelligent speech technology in epidemic prevention: Take iflytek input software in Chinese and Japanese recognition as an example. J. Phys. Conf. Ser..

[28] Le Q., Mikolov T. Distributed representations of sentences and documents. Proceedings of the 31st International Conference on Machine Learning.

[29] McInnes L., Healy J., Melville J. (2018). Umap: Uniform manifold approximation and projection for dimension reduction. arXiv.

[30] Rahman M.F., Liu W., Suhaim S.B., Thirumuruganathan S., Zhang N., Das G. (2016). Hdbscan: Density based clustering over location based services. arXiv.

[31] Blei D.M., Ng A.Y., Jordan M.I. (2003). Latent dirichlet allocation. J. Mach. Learn. Res..

[32] Bastian M., Heymann S., Jacomy M. Gephi: An open source software for exploring and manipulating networks. Proceedings of the Third International AAAI Conference on Weblogs and Social Media.

[33] Jacomy M., Venturini T., Heymann S., Bastian M. (2014). ForceAtlas2, a continuous graph layout algorithm for handy network visualization designed for the Gephi software. PLoS ONE.

[34] Madianou M., Miller D. (2013). Polymedia: Towards a new theory of digital media in interpersonal communication. Int. J. Cult. Stud..

[35] Rathnayake C., Suthers D.D. Twitter issue response hashtags as affordances for momentary connectedness. Proceedings of the 8th International Conference on Social Media & Society.

[36] Rauschnabel P.A., Sheldon P., Herzfeldt E. (2019). What motivates users to hashtag on social media?. Psychol. Mark..

[37] Daley M.F., Narwaney K.J., Shoup J.A., Wagner N.M., Glanz J.M. (2018). Addressing parents’ vaccine concerns: A randomized trial of a social media intervention. Am. J. Prev. Med..

[38] Leng A., Maitland E., Wang S., Nicholas S., Liu R., Wang J. (2021). Individual preferences for COVID-19 vaccination in China. Vaccine.

